# Cx43-Dependent Skeletal Phenotypes Are Mediated by Interactions between the Hapln1a-ECM and Sema3d during Fin Regeneration

**DOI:** 10.1371/journal.pone.0148202

**Published:** 2016-02-01

**Authors:** Jayalakshmi Govindan, Kyaw Min Tun, M. Kathryn Iovine

**Affiliations:** Department of Biological Sciences, 111 Research Drive, Iacocca B217, Lehigh University, Bethlehem, Pennsylvania, United States of America; Institute of Molecular and Cell Biology, SINGAPORE

## Abstract

Skeletal development is a tightly regulated process and requires proper communication between the cells for efficient exchange of information. Analysis of fin length mutants has revealed that the gap junction protein Connexin43 (Cx43) coordinates cell proliferation (growth) and joint formation (patterning) during zebrafish caudal fin regeneration. Previous studies have shown that the extra cellular matrix (ECM) protein Hyaluronan and Proteoglycan Link Protein1a (Hapln1a) is molecularly and functionally downstream of Cx43, and that *hapln1a* knockdown leads to reduction of the glycosaminoglycan hyaluronan. Here we find that the proteoglycan aggrecan is similarly reduced following Hapln1a knockdown. Notably, we demonstrate that both hyaluronan and aggrecan are required for growth and patterning. Moreover, we provide evidence that the Hapln1a-ECM stabilizes the secreted growth factor Semaphorin3d (Sema3d), which has been independently shown to mediate Cx43 dependent phenotypes during regeneration. Double knockdown of *hapln1a* and *sema3d* reveal synergistic interactions. Further, *hapln1a* knockdown phenotypes were rescued by Sema3d overexpression. Therefore, Hapln1a maintains the composition of specific components of the ECM, which appears to be required for the stabilization of at least one growth factor, Sema3d. We propose that the Hapln1a dependent ECM provides the required conditions for Sema3d stabilization and function. Interactions between the ECM and signaling molecules are complex and our study demonstrates the requirement for components of the Hapln1a-ECM for Sema3d signal transduction.

## Introduction

Vertebrate skeletal morphogenesis is a highly coordinated and tightly regulated process that contributes to the formation of bones of the correct size and shape. The underlying mechanisms that control the regulation of skeletal development are largely unknown. However, it is clear that proper communication and exchange of information between different cells of the skeletal tissue is crucial for proper bone formation. Gap junction mediated intercellular communication is one such mechanism that is known to contribute to skeletal development [1–3]. Intercellular communication through gap junctions involves the direct exchange of ions, second messengers and small metabolites between the cells and allows for coordinated cellular activity. It is hypothesized that gap junctional intercellular communication (GJIC) facilitates a range of functions including growth, differentiation, morphogenesis, skeletogenesis and developmental signaling [4,5]. Gap junctions are composed of oligomeric integral membrane protein subunits called connexons. Connexons from adjacent cells join to form the continuous gap junction channel that connects their cytoplasmic milieu allowing for direct exchange of low molecular weight (<1000 Da) metabolites [6]. Each connexon in turn is made up of trans-membrane protein subunits called connexins. Among the 21 different connexins known to be expressed in humans [7], *CONNEXIN43 (CX43)* is the major connexin expressed in bone cells [8]. Autosomal dominant missense mutations in human and mouse *CX43* results in the syndrome Oculodentodigital dysplasia (ODDD), characterized by craniofacial and limb abnormalities [1,2]. The *CX43*^*-/-*^ knockout mouse model exhibits severely delayed appendicular skeletal ossification and hypo-mineralization of craniofacial bones [9]. A similar set of phenotypes was observed in the targeted *CX43* knockdown chick model [10,11]. Recessive homozygous mutations in zebrafish *cx43* results in the *short fin* (*sof*^*b123*^) phenotype characterized by short fins, reduced cell proliferation and short bony fin ray segments [3]. Together, these studies reveal that Cx43-dependent GJIC exhibits conserved skeletal functions across vertebrates. The mechanisms by which *CX43* dependent mutations result in the observed skeletal phenotypes remain largely unknown.

To study the role of Cx43 during skeletal morphogenesis, we utilize the fin length mutant *sof*^*b123*^ that expresses reduced levels of *cx43* mRNA and Cx43 protein without a lesion in the coding sequence. Three additional alleles of *sof* are caused by missense mutations in the *cx43* coding sequence, which exhibit defects in GJIC using heterologous assays [12]. Based on the *sof* phenotypes of reduced cell proliferation and short segments (i.e. premature joint formation), we suggest that Cx43 regulates skeletal morphogenesis by coordinating growth (i.e., promoting cell proliferation) and patterning (i.e., inhibiting joint formation) [13,14].

To understand how Cx43-based GJIC influences tangible cellular events like proliferation and differentiation, we completed a microarray analysis to identify candidate genes that may function downstream of *cx43* [15]. Two genes identified by the microarray and subsequently validated include *semaphorin3d (sema3d)*, a secreted signaling molecule, and *hyaluronan and proteoglycan link protein 1a (hapln1a)*, an extracellular matrix (ECM) protein [15,16]. Through previous studies, we have shown that Hapln1a and Sema3d are molecularly and functionally downstream of Cx43, mediating Cx43-dependent cell proliferation and joint formation. We have also shown that *sema3d* and *hapln1a* are transcriptionally independent of each other, since knockdown of one does not affect the expression of the other [16]. However, the possibility remains that the *sema3d* and *hapln1a* gene products functionally interact.

The major function of Hapln1 is to “link” and stabilize the interaction between the glycosaminoglycan (GAG) hyaluronic acid (HA) and proteoglycans (PGs) in the ECM (Hapln1 is also called Cartilage link protein 1 or link protein/LP). HA is an unsulfated GAG composed of repeating disaccharide units of D-glucoronic acid and N-acetyl-D-glucosamine, and is synthesized in a high molecular weight form, around 10^6^−10^7^ Da. In cartilage, the majority of PGs are involved in the formation of an aggregated structure built of HA, PGs and LP (Hapln1), that are non-covalently bound [17]. Up to 100 aggrecan molecules may be associated with a single HA molecule in a newly synthesized PG aggregate, each stabilized by Hapln1 [18]. Together HA and PGs confer precise, fixed, and confined charge density imparting specific functional and structural properties to the tissue, where Hapln1 influences the spacing of the PG monomers along the length of the HA filament [17,19–22]. Other PG members include versican, neurocan and brevican, but only aggrecan and versican have been described in cartilage, and among these aggrecan has been shown to be predominantly present [23]. The mouse knockout for *CRTL1* (aka *HAPLN1*) results in perinatal lethality, dwarfism, delayed ossification, short limbs, defects in cartilage and bone development and craniofacial abnormalities [24] showing that CRTL1 is both essential and critical for proper skeletal development. In a recent study, we found that *hapln1a* knockdown in regenerating zebrafish fins causes reduced levels of HA in vivo, suggesting that the ECM becomes partially destabilized when Hapln1a is reduced [16]. However, since Hapln1a is involved in stabilizing the interaction between HA and PGs, it is possible that, in addition to HA, the PG levels could also be affected upon *hapln1a* knockdown. ECM remodeling is well known to be essential for proper skeletal development and regeneration [25–28]. Mounting evidence suggests that in addition to providing structural stability the ECM also has roles in sequestering growth factors, presenting growth factors to their receptors, and sensing and transducing mechanical signals [29,30]. Therefore, it is of interest to define how reduced Hapln1a causes Cx43-dependent skeletal phenotypes.

This report builds upon our prior studies showing that *hapln1a* knockdown results in reduced regenerate length, reduced segment length, reduced cell proliferation and reduced HA [16]. Here, we explore the possibilities whether reduced HA is sufficient to cause the observed phenotypes. As a part of these studies, we also found that aggrecan (Acan) levels are reduced following *hapln1a* knockdown and that reduced Acan also contributes to the *hapln1a* knockdown phenotypes. Finally, we provide evidence that, while *hapln1a* and *sema3d* gene transcription are independently regulated downstream of *cx43* [16], the Hapln1a and Sema3d protein products appear to interact functionally. These findings provide important new insights into the role of Hapln1a during fin regeneration and into the requirement for the ECM during Sema3d-based signal transduction.

## Materials and Methods

### Statement on the ethical treatment of animals

This study was carried out in strict accordance with the recommendations in the Guide for the Care and Use of Laboratory Animals of the National Institutes of Health. The protocols used for this manuscript were approved by Lehigh’s Institutional Animal Care and Use Committee (IACUC) (protocol identification #128, approved 11/16/2014). Lehigh University’s Animal Welfare Assurance Number is A-3877-01. All experiments were performed to minimize pain and discomfort.

### Housing and husbandry

Zebrafish are housed in a re-circulating system built by Aquatic Habitats (now Pentair). Both 3 L tanks (up to 12 fish/tank) and 10 L tanks (up to 30 fish/tank) are used. The fish room has a 14:10 light:dark cycle and room temperature (RT) varies from 27–29°C [31]. Water quality is automatically monitored and dosed to maintain conductivity (400–600 μs) and pH (6.95–7.30). Nitrogen levels are maintained by a bio filter. A 10% water change occurs daily. Recirculating water is filtered sequentially through pad filters, bag filters, and a carbon canister before circulating over UV lights for sterilization. Fish are fed three times daily, once with brine shrimp (hatched from INVE artemia cysts) and twice with flake food (Aquatox AX5) supplemented with 7.5% micropellets (Hikari), 7.5% Golden Pearl (300–500 micron, Brine Shrimp direct), and 5% Cyclo-Peeze (Argent).

### Experimental procedures and animals

Wild-type C32 zebrafish (*Danio rerio*) and *Tg(hsp70*:*sema3dgfp)* (generously provided by Mary C. Halloran) were used in this study. Fish were anaesthetized in 0.1% tricaine and caudal-fin amputations were performed at 50% level. Fin regeneration was allowed to proceed until the desired time period (3–7 days post amputation [dpa]) depending on the type of experiment. The regenerated fins were harvested at the required time point after anaesthetizing the fish. Whole mount in situ hybridization, immunofluorescence and histochemical staining were performed on regenerating fins. Descriptions of each of these methods follow.

### *In situ* hybridization on whole mount fins and cryo-sections

For each gene under study, a minimum of 4–5 fins were used per trial and 3 independent trials were performed. Fins were fixed overnight in 4% paraformaldehyde (PFA) in PBS and dehydrated in 100% methanol and stored at -20°C until use, for in situ hybridization (ISH). RNA probes were generated from PCR amplified linear DNA in which the reverse primer contained the T7 RNA polymerase binding site. The primers used in this study for ISH are summarized in Table 1. Digoxygenin (DIG) labeled RNA probes were synthesized using DIG labeling mix (Roche) and in situ hybridization on regenerated whole fins was carried out following standard protocols [15,16].

**Table 1:** Primer Sequences.

Gene	Primer Sequence for ISH
***hapln1a***	FP-ggttctccgcttggcagcg
	RT7-**taatacgactcactataggg**gcccatccctgcctaagacc
***sema3d***	FP-cgaagtgtagtaccatttacg
	RT7-**taatacgactcactataggg**tatgaggatcatatgtcc
***has1***	FP-gaaatttgtgtctctggtccgagc
	RT7-**taatacgactcactataggg**gacctaaggggccgc
***has2***	FP-gttgggacgacactgttcgg
	RT7-**taatacgactcactataggg**ctcgattggtcagatggcgg
***has3***	FP-ggtgcggatcttcatcaccacc
	RT7-**taatacgactcactataggg**ggtctggtggtaccagggc
***Acana***	FP-cttccaggacaacacagtcaacg
	RT7-**taatacgactcactataggg**cttcatcgcctgtttcagagtagc
***Acanb***	FP-cccatgattctggcacctaccg
	RT7-**taatacgactcactataggg**gcatagcgcccagattcagc

The RNA polymerase T7 binding site in reverse primers is highlighted in bold.

F = Forward primer; RT7 = Reverse primer

For in situ on cryo-sections, fins were fixed overnight with 4% PFA in PBS after harvest. After a brief methanol wash, fins were dehydrated in 100% methanol and stored at -20°C until use. Before sectioning, fins were sequentially rehydrated in a methanol- PBS series of washes and then were embedded in 1.5% agarose/5% sucrose dissolved in PBS and equilibrated overnight in 30% sucrose. Fins were mounted in OCT and cryo-sectioned (15 μm sections) using a Reichertaˆ Jung 2800 Frigocut cryostat. Sections were collected on Superfrost Plus slides (Fisher) and allowed to air dry overnight at room temperature. Following this in situ on cryo-sections were performed following standard protocols as described [15,16,32]. Finally sections were mounted in 100% glycerol and examined on a Nikon Eclipse 80i microscope.

### Morpholino mediated gene knockdown in regenerating fins

All MOs used in this study were fluorescein tagged, obtained from Gene Tools, LLC and used at a final concentration of 1mM for injection, unless otherwise described. The sequences for MOs used in this study can be found in Table 2. For each gene under study two MOs, one targeting translation initiation and the other targeting splicing were used. The effect of the MO was tested either by histochemistry or by RT-PCR as described below. Injection and electroporation were carried out as described previously [16,33]. Briefly, 3 dpa fish were anesthetized and approximately 50nl of MO (targeting morpholino [MO] or mismatch control morpholino [MM]) or standard control MO 5’ CCTCTTACCTCAGTTACAATTTATA 3’ was injected into either the dorsal or the ventral half of the tail fin. The un-injected half of the fin served as the internal control. Following injection, the entire fin was electroporated using a CUY21 Square Wave electroporator (Protech International Inc). MO-positive fish were selected 24 hpe (hours post electroporation) by examination under a fluorescence microscope. The knockdown fins were evaluated for regenerate length, segment length, histone-3-phosphate (H3P) analysis, alizarin red staining, immuno-fluorescence and histochemical experiments. The procedure for each analysis is described separately. For each MO (i.e., targeting or mismatch) 6–8 fish were used. Reproducibility was confirmed by testing the MO in three independent experiments. Statistical significance was determined using the student's *t*-test (P<0.05).

**Table 2:** Morpholino Sequences.

Gene	Morpholino
***hapln1a***	MO-gccacagaaaacagagcaatcatct
	MM-gccagacaaaagagaccaatgatct
***sema3d***	MO-tgtccggctcccctgcagtcttcat
	MM-tgtgccgctgccctccactcttcat
***has1***	MO1-tctttaagactggcttgaggtccat
	MO2-gtcacatacctgattgaaaaataca
	MM-tcttaaacagtggcttcacgtccat
***has2***	MO1-gctgaccgctttatcacatctcatc
	MO2-tgaaagagagacagactcacctgta
	MM-gctcaccccttaatcagatgtcatc
***Acanb***	MO1-acagcaggagccaaatcaaagacat
	MO2-gggcttgatttaaagtgccttacct

MO1 = ATG-blocking morpholino.

MO2 = splice-blocking morpholino.

MM = Control morpholino with 5 mismatch pairs.

### Rescue of *hapln1a* knockdown phenotypes with *sema3d* overexpression

We used three groups of fish for these experiments. Groups 1 and 2 were positive for the transgene, Tg(*hsp70*:*sema3d-gfp*), while Group 3 fish were negative for the transgene. Knockdown of *hapln1a* was performed on 3 dpa fins as described above. Four hours after *hapln1a* knockdown, heat shock was performed for one hour at 37°C (Groups 1 and 3). Alternatively, Group 2 fish were positive for the transgene but were not exposed to heat shock. Induction of transgene expression in Group 1 animals was confirmed after 24 hours of heat shock by screening for GFP-positive fins. For regenerate and segment length analyses the fins were harvested 4 dpe (days post electroporation) and data analysis was performed as described below. For each experiment a minimum of 6–8 fins were used per trial and 3 independent trials were performed. Statistical significance was determined using the student's *t*-test (P<0.05).

### Fin regenerate length, segment length and H3P analysis

For each type of analysis, minimum of 6–8 fins were used per trial and 3 independent trials were performed. Statistical significance was determined using the student's *t*-test (P<0.05). Images were collected on a Nikon SMZ1500 dissecting microscope using a Nikon DXM1200 digital camera unless otherwise stated. Measurements were performed using Image Pro software.

Fin regenerate length analysis was performed as described [15]. Briefly, fins were harvested at 4 dpe, fixed and stored in methanol until use at -20°C. The measurements were taken from the longest fin ray (i.e. the third ray from either the dorsal or ventral end) that was previously established as a standard [34]. Fin regenerate length was measured using Image Pro Software from the amputation plane (clearly visible in bright field) to the tip of the fin. To evaluate the phenotypic effect of MO based knockdown experiments on fin regenerate length, the targeting MO or control MM injected side of each fin is compared to its un-injected side by the percent similarity method as described [32]. Briefly, the percent similarity for each fin is calculated as ([length of the injected side in μm / length of the un-injected side in μm] X 100). Values close to 100% indicate that the injected MO has no effect on the phenotype whereas a value less than 100% indicate that the MO has an effect on the observed phenotype. The mean of percent similarity for the MO treated experimental group and the corresponding MM treated control group were estimated and compared. Percent similarity of greater than 100% reflects the fact that the experimental side can be measurably larger than the control un-injected side. Statistical significance was determined using the student's *t*-test (P<0.05).

Segment length analysis was performed on calcein stained fins. Calcein staining was performed on live fish as described [35]. Briefly, fish were permitted to swim in 0.2% calcein for 10 min, followed by 10 min in fresh water. Fish were anesthetized as described above and fins were imaged at 4X. For segment length, the distance between the first two newly formed joints following amputation was measured in the third fin ray from either the dorsal or ventral end [34]. Segment length was measured using Image Pro Software. To evaluate the phenotypic effect of MO based knockdown experiments on segment length, the percent similarity method was used as described above.

For H3P analysis, the fins were harvested 1 dpe, fixed overnight in 4% paraformaldehyde (PFA) in PBS and dehydrated in 100% methanol and stored at -20°C until use. Cell proliferation analysis was performed by counting number of mitotic cells by H3P immuno-staining as described [15]. H3P positive cells were counted from within the distal most 250 μm of each fin above the third fin ray from either the dorsal or ventral end [34]. To evaluate the phenotypic effect of MO based knockdown experiments on cell proliferation, the percent similarity method was used as described above. For cell proliferation, the percent similarity for each fin is calculated as ([# H3P positive cells per 250 μm^2^ of injected side / # H3P positive cells per 250 μm^2^ of un-injected side] X 100).

### Alizarin red staining

For alizarin red staining, the fins were harvested 4 dpe, fixed overnight in 4% paraformaldehyde (PFA) in PBS and dehydrated in 100% methanol and stored at -20°C until use. Fins were rehydrated through a decreasing methanol series as described for ISH. Following that fins were bleached for 30 minutes in 0.8% KOH, 0.6% H_2_O_2_ (should be prepared and used within one week). Subsequently, fins were washed twice with water and then washed for 1hr in a saturated Alizarin Red solution containing 1% KOH [36] followed by 30 minutes wash in water and mounted in glycerol for imaging. The extent of mineralization was calculated as the ratio of the zone of mineralization (extent of detectable alizarin red staining length) to the total regenerate length. To evaluate the phenotypic effect of MO based knockdown experiments on alizarin red staining, the % similarity method was used as described above. The % similarity for each fin is calculated as ([ratio of {regenerate length: alizarin red staining length} of the injected side / ratio of {regenerate length: alizarin red staining length}of un-injected side] X 100). The mean of % similarity for the MO treated experimental group and the corresponding MM treated control group were estimated and compared, and the statistical significance between the groups was determined using two tailed unpaired student's *t*-test (P<0.05). For each experiment minimum of 6–8 fins were used per trial and 3 independent trials were performed.

### Cryosectioning for immunofluorescence and histochemistry

Fixed and embedded fins were mounted in OCT and cryosectioned (15 μm sections) using a Reichertâ Jung 2800 Frigocut cryostat. Sections were collected on Superfrost Plus slides (Fisher) and allowed to air dry O/N at RT. Sections can be stored at –20°C for up to a year. The slides were stored at –20°C for at least one day before starting the experiment. For all experiments using cryosections, the slides were brought to RT for one hour. Sections were circled using a marking pen (ImmEdge Pen H-4000; PAP pen, VWR Laboratories). To confirm reproducibility, minimum of 3–5 different fins were used for each experiment and approximately 8–12 sections per fin were analyzed.

### Acan Immunofluorescence

For detection of Acan, fins were fixed for 10 min with 2% PFA at RT followed by three 10 min washes with 1X PBS, and then embedded in 1.5% agarose/5% sucrose in PBS and equilibrated in 30% sucrose in PBS and sectioned as described above. Acan immuno-staining was performed as described earlier [37]. Briefly, the sections were rehydrated twice for 10 min in PBS at RT, followed by antigen unmasking by digestion with chondroitinase. First, the slides were incubated with the chondroitinase treatment buffer (50mM Tris, 60mM sodium acetate, 0.02% BSA, pH 8.0) at 37°C for 5 min, followed by deglycosylation using chondroitinase ABC enzyme (Sigma-C2905, final concentration 0.05U in the treatment buffer) for 2 h at 37°C. Following that, slides were washed with block for 10 min at RT. Then, sections were blocked for another one hour at RT, incubated with Mouse anti-aggrecan (BC-3) (Thermo Scientific-MA3-16888) primary antibody O/N at 4°C. Following incubation with primary antibodies, sections were washed three times in block (15 min each), incubated at RT for one hour with secondary antibody goat anti-mouse Alexa-663 (Invitrogen, 1:200) or goat anti-mouse Alexa-568 (Invitrogen, 1:200) and washed again three times in block (15 min each). Sections were next incubated with propidium iodide (final concentration 0.01mg/ml in block) or To-Pro-3-iodide (Life Technologies, 1:1000) for 30 min at RT, followed by a quick wash with distilled water.

### Sema3d Immunofluorescence

For detection of Sema3d, fins were fixed for 30 min with 2% PFA at RT followed by three 10 min washes with 1X PBS, and then embedded in 1.5% agarose/5% sucrose in PBS and equilibrated in 30% sucrose in PBS and sectioned as described above. For Sema3d immuno-staining, the sections were rehydrated twice for 10 min in PBS followed by antigen retrieval. Slides were transferred to coplin jars containing 10 mM sodium citrate (pH = 6) and incubated at 99.5°C water bath for 10 minutes. The slides were allowed to cool down to room temperature and again washed in 1X PBS (2X, five minutes each). Following that slides were washed twice with block (2% BSA, 0.1% TritonX 100 in PBS). Then, sections were blocked for another one hour at RT and then incubated in primary antibody, Rabbit anti-Sema3d (Sigma-SAB1402064, 1:500) or Mouse anti-EGFP (Clonetech, 1:1000) O/N at 4°C. Following incubation with primary antibodies, sections were washed three times in block (15 min each), incubated at RT for one hour with secondary antibody goat anti-rabbit Alexa-663 (Invitrogen, 1:200) or goat anti-mouse Alexa 488 and washed again three times in block (15 min each). Sections were next incubated with propidium iodide (final concentration 0.01mg/ml in block) or DAPI (1:1000) for 30 min at RT, followed by a quick wash with distilled water.

### Histo-chemical analysis of HA

For detection of HA, fins were fixed overnight (O/N) with 4% PFA in PBS. After a brief methanol wash, fins were dehydrated in 100% methanol and stored at –20°C until use. Before sectioning, fins were sequentially rehydrated in a methanol-PBS series of washes and then were embedded in 1.5% agarose / 5% sucrose in PBS and equilibrated in 30% sucrose in PBS and then sectioned as described above. HA was detected as described [16]. Briefly the sections were rehydrated twice for 10 min in PBS followed by two washes with block. Then, sections were blocked for another one hour at RT, incubated with biotinylated hyaluronic acid binding protein (bHABP-Calbiochem-385911, 1:100) O/N at 4°C. The sections were washed three times in block (15 min each), incubated at RT for one hour with streptavidin-Alexa-546 conjugate, (Invitrogen, 1:200) and washed again three times in block (15 min each). Sections were next incubated with propidium iodide (final concentration 0.01mg/ml in block) or To-Pro-3-iodide (Life Technologies, 1:1000) for 30 min at RT, followed by a quick wash with distilled water. Then the slides were blotted dry and mounted for imaging as described above.

### Confocal microscopy

The slides were blotted dry and mounted with vectashield for imaging. Confocal microscopy was used to image the sections using a 40×/1.3 numerical aperture objective on an inverted microscope (Axiovert 200 M; Carl Zeiss, Jena, Germany) equipped with an LSM510 META scan head (Carl Zeiss). Argon ion and HeNe lasers were used to generate the 488 and 543/633 lines used for excitation, and pinholes were typically set to 1–1.5 Airy units. Images were exported as TIFF files and printed using Photoshop.

## Results

### Components of Hapln1a-ECM are expressed in the regenerating fin

**Fig 1:**
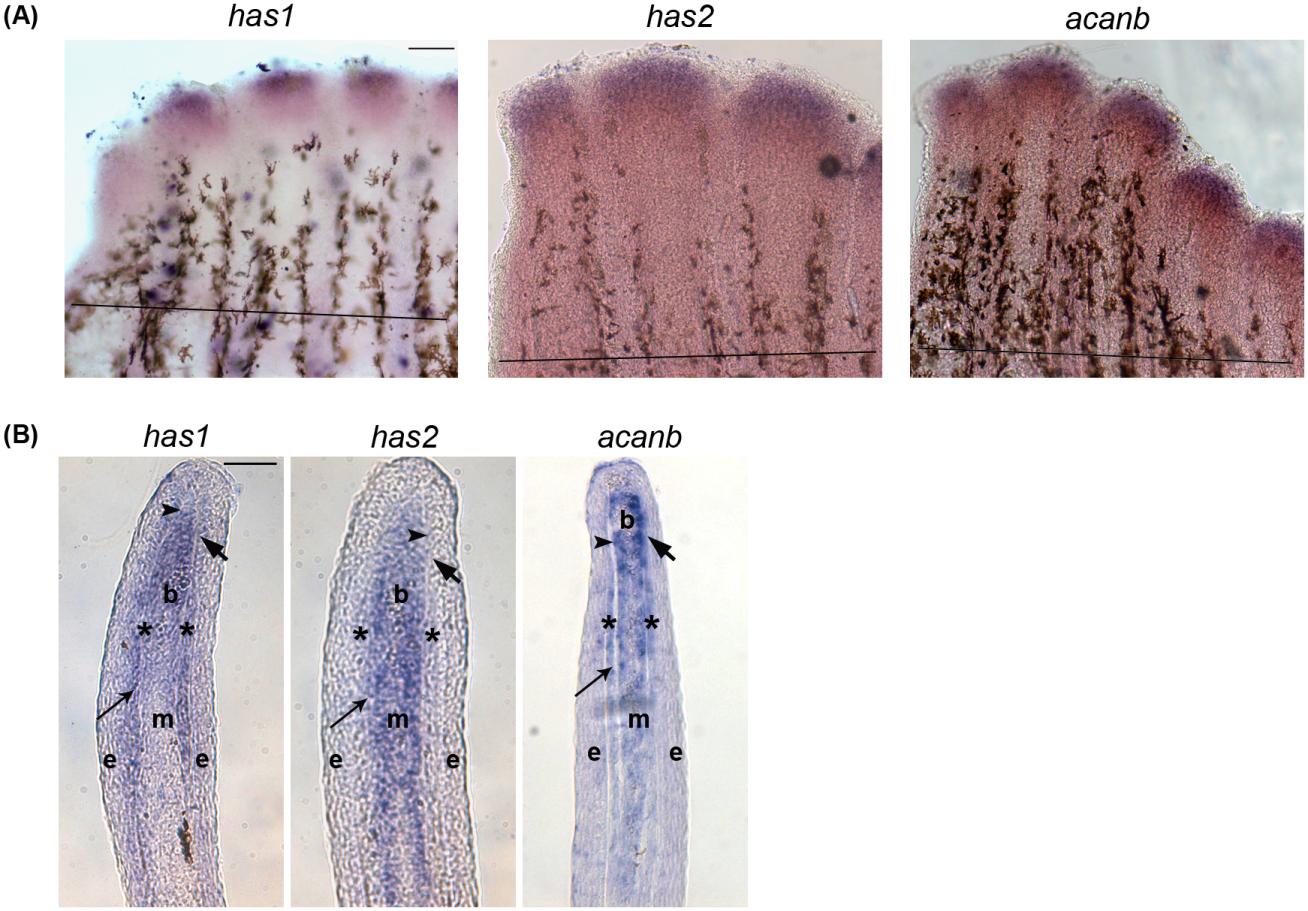
*In situ* hybridization showing the expression of Hapln1a-ECM components on 5 dpa regenerating fins. (A) Whole mount *in situ* hybridization of components of the Hapln1a-ECM. The genes *has1*, *has2*, and *acanb* are expressed during fin regeneration. The amputation plane is indicated by a black line in all the panels. Scale bar represents 100μm. (B) *In situ* hybridization on a WT 5 dpa cryo-section reveals compartmental expression of *has1*, *has2*, and *acanb*. Blastema (b), mesenchyme (m), and skeletal precursor cells (*). The thick arrow identifies the basal layer of the epidermis, which underlies the epidermis (e). The thin arrow identifies lepidotrichia and the arrowhead identifies the actinotrichia. Scale bar represents 50μm.

In a recent study, we showed that *hapln1a* knockdown results in Cx43 dependent phenotypes: reduced fin regenerate length, reduced segment length and reduced cell proliferation [16]. Since Hapln1a links HA with PGs we were interested in the expression of genes responsible for synthesis of HA and in the expression of PGs. HA is synthesized by hyaluronic acid synthases (Has) and Acan is a major bone associated PGs involved in matrix organization [38]. We performed whole mount in situ hybridization on WT 5 days post amputation (dpa) regenerating fins to determine which of the HA synthesizing enzymes (*has1*, *has2* and *has3*) and which of the skeletal PGs (i.e, *acana* and *acanb*) are expressed. Of the three different HA synthesizing enzymes, *has1* and *has2* are expressed in the regenerating fin. Of the duplicate copies of *acan*, only *acanb* is expressed in the regenerating fin (Fig 1A). To assess in which tissues these genes are expressed, we completed in situ hybridization on cryosections of 5 dpa regenerating fins (Fig 1B). The medial mesenchyme consists of both distally located slowly proliferating cells and proximally located rapidly proliferating cells [39]. The lateral mesenchyme consists mainly of skeletal precursor cells, which includes the precursors of bone-forming osteoblasts and joint-forming cells [40]. Several epidermal layers surround the mesenchyme, and the basal layer of epidermis resides closest to the mesenchyme. We find that *has1* is expressed in the medial and lateral mesenchyme, as well as in the basal layer of the epidermis, while *has2* is expressed in the medial mesenchyme (and not in the lateral mesenchyme). The *acanb* mRNA is expressed throughout the mesenchyme, consistent with the localization of the Acan protein [37].

### Reduced HA levels contribute to the Hapln1a mediated phenotypes

**Fig 2:**
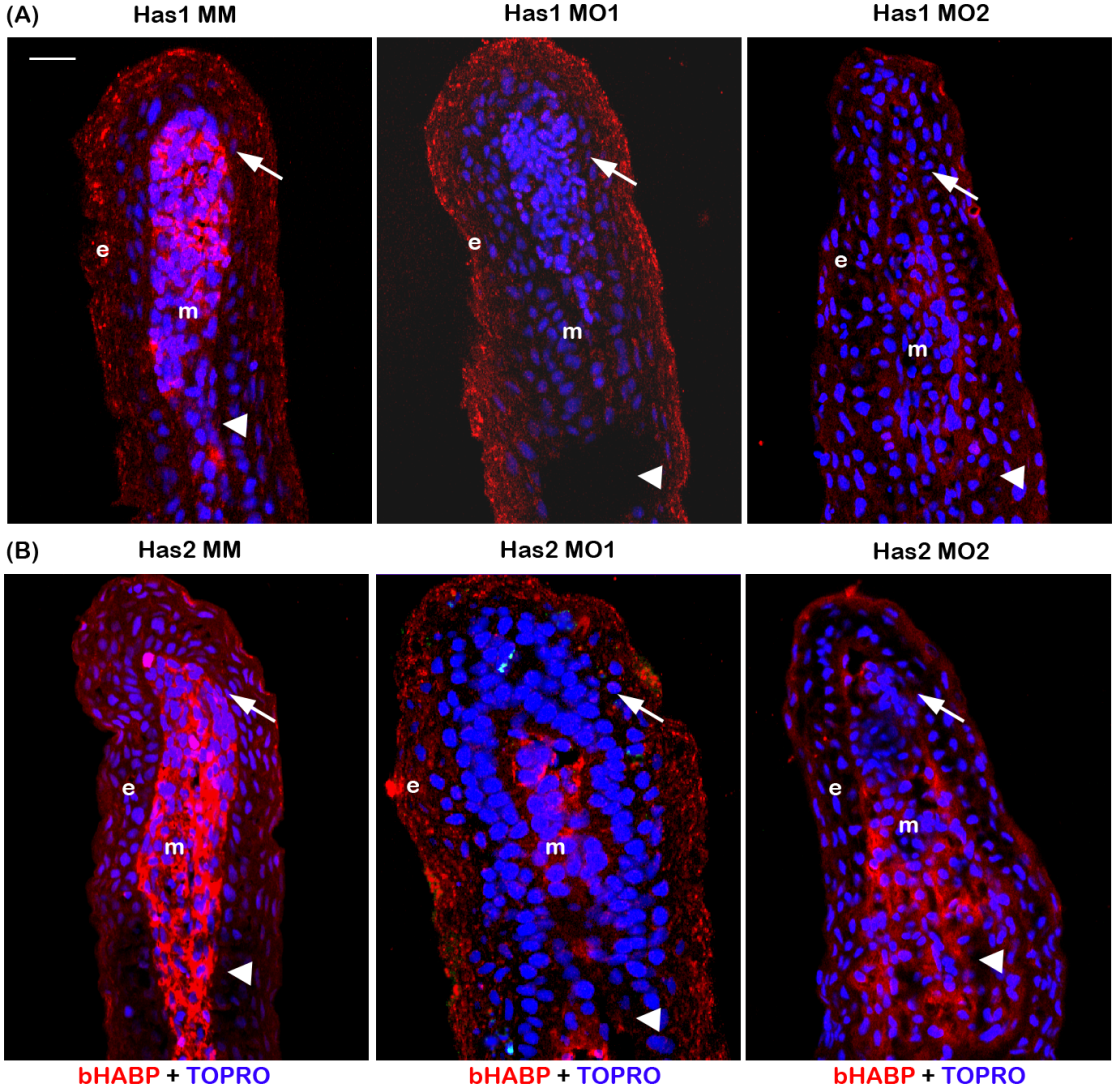
Morpholino mediated knockdown of *has1* and *has2* results in reduced HA. (A) Morpholino mediated knockdown of *has1* results in reduced HA. Longitudinal section of fin rays treated with *has1* control morpholino (Has1 MM) and *has1* target morpholinos (Has1 MO1 and Has1 MO2). (B) Morpholino mediated knockdown of *has2* results in reduced HA. Longitudinal section of fin rays treated with *has2* control morpholino (Has2 MM) and *has2* target morpholinos (Has2 MO1 and Has2 MO2). HA is detected by histochemical staining using biotinylated HABP and Streptavidin-Alexa 546. TO-PRO is used as the counterstain and detects DNA (blue). Compared to Has1 MM and Has2 MM treated fins, Has1 MO and Has2 MO treated fins exhibit reduced staining for HA. Arrow identifies the basal layer of epithelium; arrow head identifies the bone; m, mesenchyme; e, epithelium. Scale bar represents 20 μm.

Because *hapln1a* knockdown results in reduced HA [16], we hypothesized that manipulating the HA levels by targeting the HA synthesizing enzymes should recapitulate the *hapln1a* knockdown phenotypes. Since it is likely that homozygous mutations in genes required for the Hapln1a-ECM will be essential, or will cause serious global skeletal defects, we rely on morpholino (MO) based knockdown for evaluation of gene function in the regenerating fin. For targeted gene knockdown, we use two independent non-overlapping MOs for each gene (one ATG-blocker and one splice blocker). As controls, we use either paired 5 mismatch (MM) non-targeting control MOs or the standard control MO. Further, we also confirm that the target is reduced following knockdown. In order to manipulate the HA levels, we completed MO mediated knockdown of the HA-synthesizing enzymes *has1* and *has2* in WT regenerating fins. All MOs used in this study are fluorescein tagged, permitting the confirmation of cellular uptake. Following MO microinjection and electroporation at 3 dpa, MO positive fish were screened 24 hours post electroporation (hpe) by detection of fluorescein positive cells. We evaluated the effect of the both ATG-blocking and splice-blocking MOs on HA synthesis for the HA synthesizing enzymes (i.e. *has1 and has2*). MO or MM positive fins were harvested 24 hpe and processed for immuno-histochemical analysis for HA using biotinylated HA binding protein (bHABP). During early regeneration (i.e. 3–5 dpa), HA is predominantly upregulated in epidermis, in the proximal mesenchyme of the regenerate, and in the mesenchyme of the stump tissue [16,37]. Histochemical analysis for HA on *has1* and *has2* knockdown fins revealed reduced levels of HA compared to the MM treated fins, indicating that HA synthesis has been successfully down regulated upon knockdown of these synthesizing enzymes (Fig 2). We did not evaluate co-localization of the fluorescein-MO and HA since processing the fins for detection with the bHABP limited detection of the fluorescein. However, we have shown previously that the fluorescein MO is found in all tissue compartments [40]. Because the MO is injected into the mesenchyme, the highest knockdown is typically observed there (although knockdown in the epidermis can sometimes also be detected). The finding that HA is reduced following knockdown demonstrates the specificity of independent MOs targeting *has1* and *has2* gene function.

**Fig 3:**
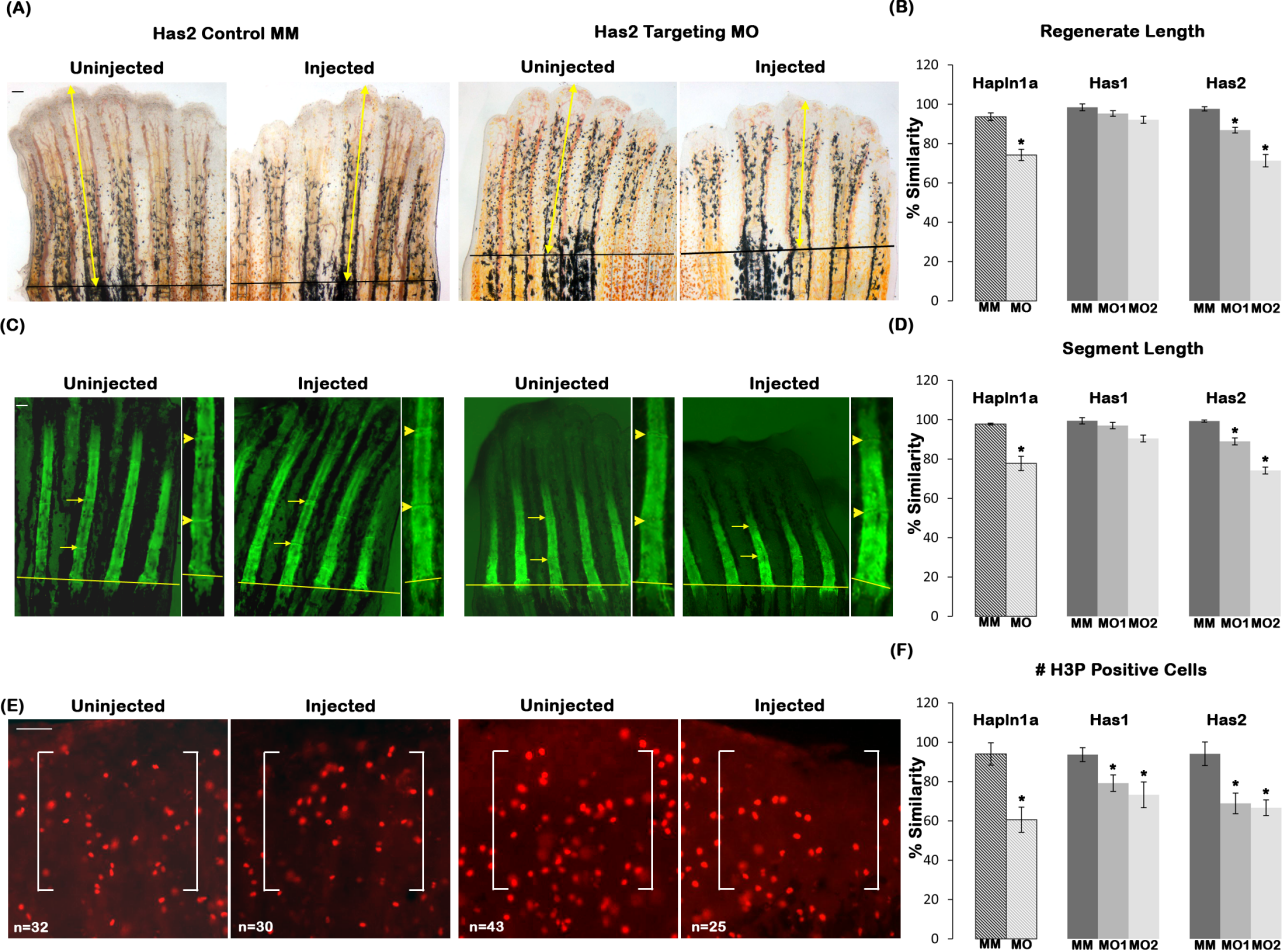
Reduced HA contributes to Hapln1a knockdown phenotypes. (A) Representative images showing regenerate length for Has2 MM and Has2 MO treated fins. Total regenerate length was evaluated by measuring the distance between the amputation plane (black line) to the distal end of the fin using the 3^rd^ fin ray (marked by yellow arrows). (B) Bar graph shows that regenerate length is significantly reduced upon *has2* knockdown but not in *has1* knockdown using two independent MOs for each gene (MO1 and MO2). The hashed bars indicate the extent of *hapln1a* knockdown effect compared to *has1* and *has2* knockdowns. (C) Representative calcein stained fins showing segment length for Has2 MM and Has2 MO treated fins. Segment length was evaluated by measuring the distance between first two joints in the regenerate (marked by yellow arrows). An enlarged inset for the measured segment is provided and the joints are indicated by yellow arrow heads. (D) Bar graph shows that segment length is significantly reduced upon *has2* knockdown but not in *has1* knockdown using two independent MOs for each gene (MO1 and MO2). The hashed bars indicate the extent of *hapln1a* knockdown effect compared to *has1* and *has2* knockdowns. (E) Representative H3P stained fins showing H3P positive cells for Has2 MM and Has2 MO treated fins. H3P positive cells were counted within a defined area (marked by white brackets). (F) Bar graph shows that cell proliferation is significantly reduced upon *has1* and *has2* knockdown using two independent MOs for each gene (MO1 and MO2). The hashed bars indicate the extent of *hapln1a* knockdown effect, compared to *has1* and *has2* knockdowns. Students t-test was performed (p<0.05) to determine significance, and the error bars indicate standard error of the mean. Scale bar is 50 μm in all panels.

We next evaluated the effects of the *has1* and *has2* knockdowns on Cx43-Hapln1a dependent phenotypes using the ATG-blocking and the splice-blocking targeting MOs, and comparing to control-treated fins. For analysis, either the target MO or the control MM is injected into fin rays in one half of the fin, and the other half is un-injected. Next, the injected side was compared with the respective un-injected side and the percent similarity was determined. This method reduces the effect of fin to fin variation. High similarity between the injected and un-injected side (i.e. close to 100%) indicates little or no effect of the MO treatment, whereas values with low similarity (i.e. less than 100%) indicates that the MO had an effect on treatment side. Fin length was measured at 4 dpe as the distance from the amputation plane to the distal end of the regenerate (Fig 3A). We observed that knockdown of *has1* had no effect on regenerate length, whereas *has2* knockdown resulted in significant reduction in regenerate length compared to the MM control (Fig 3B). The segment length was measured at 4 dpe by calcein staining as the distance between the first two joints distal to the amputation plane (Fig 3C). Similar to regenerate length, *has1* knockdown had no effect on segment length; whereas *has2* knockdown resulted in significant reduction in segment length phenotype (Fig 3D). Because *has1* is expressed in skeletal precursor cells but *has2* is not, this finding suggests that the HA produced from the nearby medial mesenchyme contributes to segment length. The effect of the knockdown of these enzymes on cell proliferation was evaluated by counting the number of mitotic cells following immuno-fluorescence for H3P at 1 dpe (Fig 3E). For cell proliferation we found that *has1* knockdown resulted in ~ 20–30% reduction, whereas *has2* knockdown had a reduction of about 35–38%. Therefore, knockdown of both enzymes resulted in significant reduction in cell proliferation compared to the control MM treated fins (Fig 3F), and we note that both *has1* and *has2* are expressed in the proliferating cells. However, the *has2* knockdowns are stronger than the *has1* knockdowns for all phenotypes.

**Fig 4:**
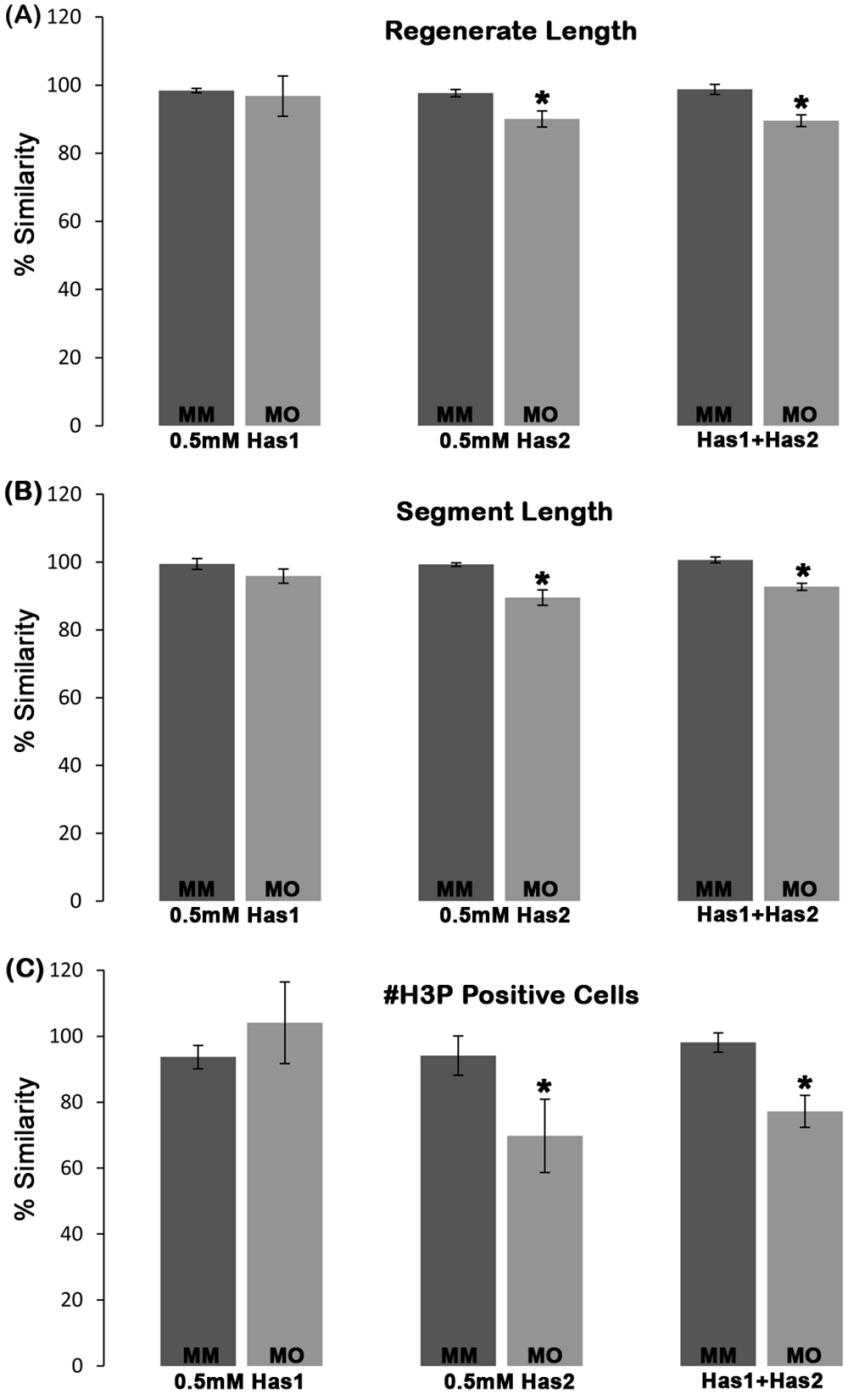
Double knockdown experiments suggest that HA produced by *has1* and *has2* may be functionally distinct. Double knockdown of *has1* and *has2* was performed by injecting/electroporating combined 1 mM stocks of each ATG-blocking MO (or of each MM MO), resulting in 0.5 mM concentrations of the each MO. This was compared to single knockdowns of 0.5 mM concentrations for each targeting or MM MO. Bar graphs of percent similarity show that the double knockdown does not result in synergistic defects for regenerate length (A), segment length (B), or cell proliferation (C). Students t-test was performed (p<0.05) to determine significance, and the error bars indicate standard error of the mean. None of the double knockdowns were statistically different from the knockdown of *has2* alone (i.e. which is the only single knockdown to show phenotypic effects).

Since *has1* and *has2* knockdown both lead to reduced, but not completely absent levels of HA, it is unclear why *has2* knockdown influences regenerate length and segment length and *has1* knockdown does not. One possibility is that the HA produced by Has1 and Has2 has sufficiently distinct properties that mediate distinct functions. All Has enzymes synthesize HA of similar composition, but differ in their rate of synthesis, molecular weight of HA, and ease with which HA is released from the cell surface [41]. Moreover, others have shown that neither the Has1 nor the Has3 enzymes can replace the function of Has2 during heart morphogenesis [42], even though all three Has enzymes are expressed [43]. Therefore, it is possible that the HA synthesized by Has2 could be functionally distinct. Alternatively, the effects of the *has1* knockdown may be diminished due to potential redundancy with *has2*. To distinguish these possibilities, we completed double knockdown of Has1 and Has2. Double knockdowns were performed by combining both *has1* and *has2* ATG-blocking MOs (i.e. 0.5mM of each MO) and comparing to single knockdowns of *has1* and *has2* at 0.5 mM MO concentrations. For the double knockdowns and each single knockdown, either the targeting MO or the control MM injected sides were compared with the respective un-injected side and the percent similarity was determined as described earlier (Fig 4). The double knockdown failed to demonstrate synergy of the two MOs, which argues against functional redundancy of the *has1* and *has2* gene products. Rather, our findings are consistent with a model that the HA produced by *has1* and *has2* is functionally distinct.

To determine if reduced HA is sufficient to mediate Hapln1a effects, we compared the reduction in regenerate length, segment length and cell proliferation of *has2* knockdown with *hapln1a* knockdown that we determined previously [16]. The effect of *hapln1a* knockdown levels are represented as hashed bars in Fig 3B, 3D and 3F. Interestingly, we find that *hapln1a* knockdown alone resulted in a much stronger effect on the level of cell proliferation than the knockdown of either *has1* or *has2*. Therefore, in addition to HA there could be other Hapln1a-ECM components contributing to Hapln1a-Cx43 dependent phenotypes that account for the stronger *hapln1a* knockdown phenotype.

### Acan contributes to the observed *hapln1a* knockdown skeletal phenotypes

**Fig 5:**
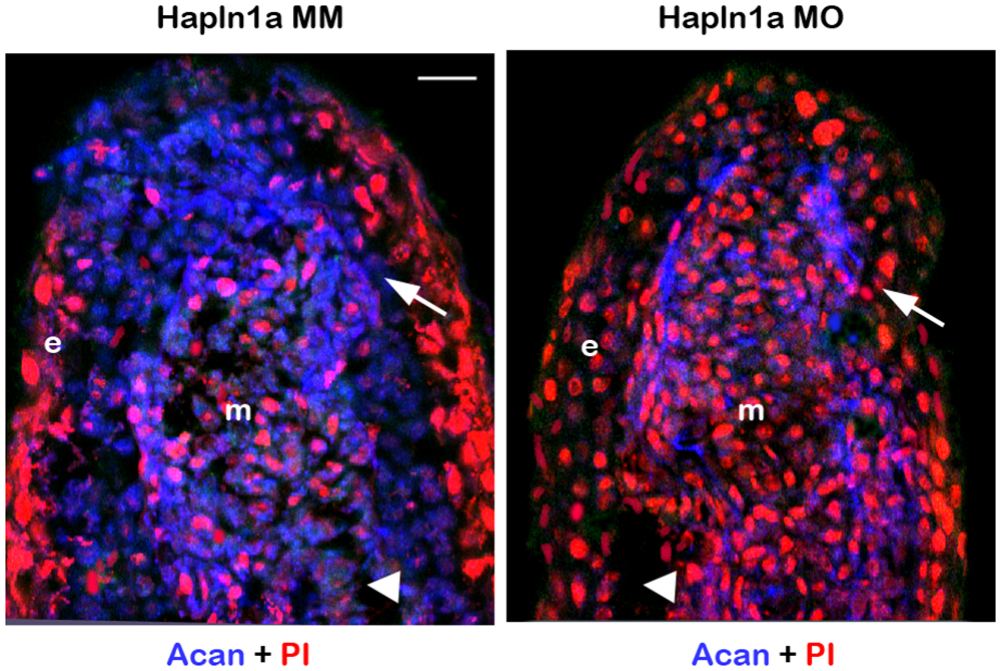
Morpholino mediated knockdown of *hapln1a* results in reduced Acan protein levels. Longitudinal section of fin rays treated with *hapln1a* control morpholino (Hapln1a MM) and *hapln1a* targeting morpholino (Hapln1a MO). Immuno-staining for Acan (blue) and counterstained for nuclei with Propidium Iodide (PI, red). Compared to the control Hapln1a MM treated fins, Hapln1a MO treated fins show reduced staining for Acan. Arrow identifies the basal layer of epithelium; arrow head identifies the bone; m, mesenchyme; e, epithelium. Arrow identifies the basal layer of epithelium; arrow head identifies the bone; m, mesenchyme; e, epithelium. Scale bar represents 20 μm.

**Fig 6:**
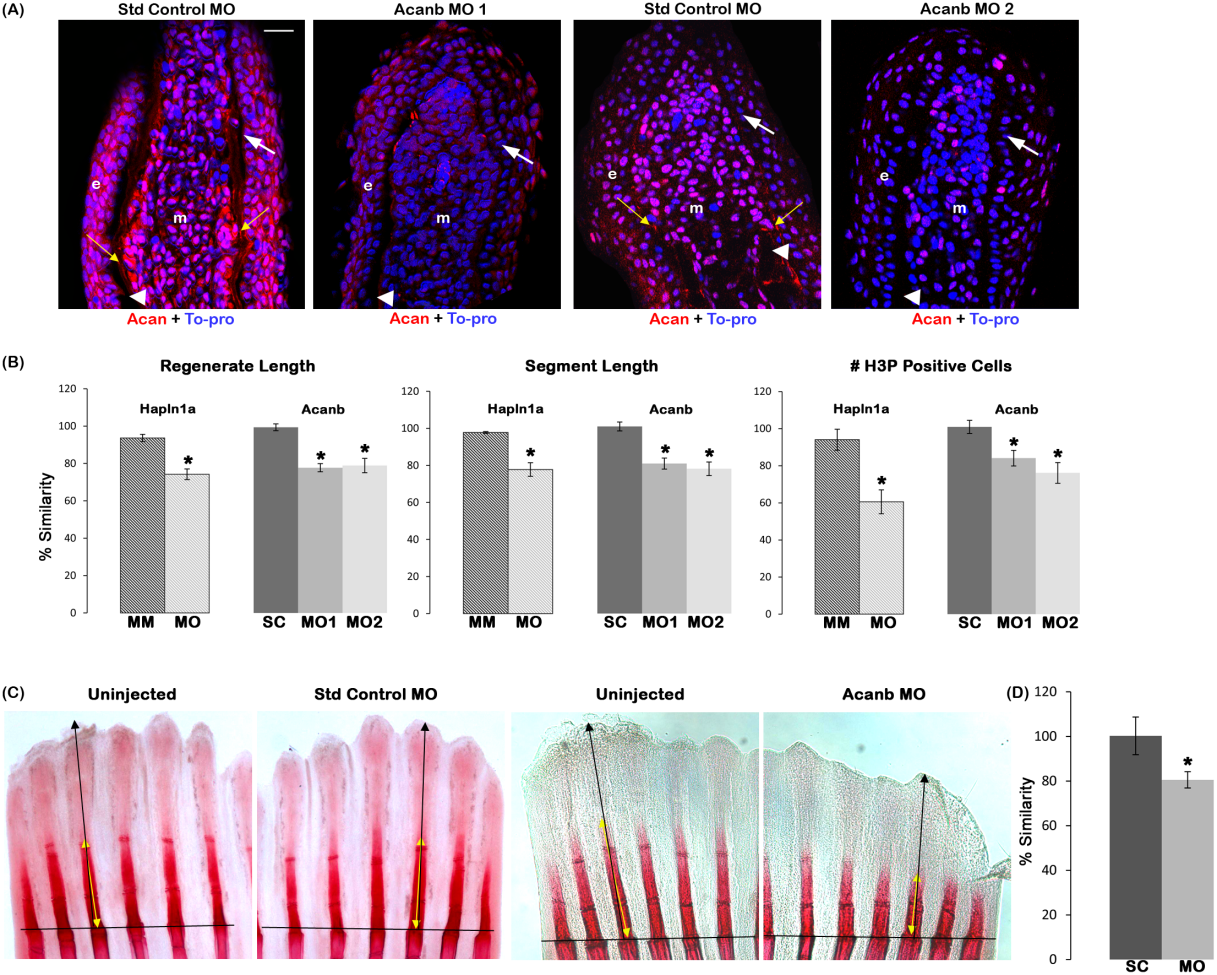
Reduced Acan contributes to Hapln1a knockdown phenotypes. (A) Longitudinal section of fin rays treated with standard control morpholino (Standard control MO) and *acanb* ATG-blocking morpholino (Acanb MO1) or splice blocking morpholino (Acanb MO2). Immuno-staining for Acan (red) and counterstained for nuclei with To-pro (blue). Compared to the standard MO treated fins, Acanb MO treated fins show reduced staining for Acan. White arrow identifies the basal layer of epithelium; yellow arrow identifies Acan staining in the lepidotrichia, arrow head identifies the bone; m, mesenchyme; e, epithelium. Scale bar represents 20 μm. (B) Bar graph shows that regenerate length, segment length and cell proliferation are significantly reduced upon *acanb* knockdown using both MO1 and MO2. The mean of percent similarity for the MO treated experimental group and the corresponding control group were estimated and compared. Statistical significance was determined using the student's *t*-test (P<0.05) and the error bars represent standard error of mean. The hashed bars indicate the extent of *hapln1a* knockdown effect compared to *acanb* knockdown. (C) Representative alizarin red stained fins showing extent of bone calcification. The black line indicates the amputation plane. (D) The extent of mineralization was calculated as the ratio of the zone of mineralization (extent of detectable alizarin red staining length) to the total regenerate length. The mean of percent similarity for the MO treated experimental group and the corresponding control group were estimated and compared, and the statistical significance between the groups was determined using two tailed unpaired student's *t*-test (P<0.05) and the error bars indicate the standard error of mean.

Acan is a major PG associated with bones [38]. In addition to HA, we hypothesized that the Hapln1a associated Acan may also contribute to Hapln1a mediated skeletal phenotypes. We first determined if Acan protein levels are reduced in *hapln1a* knockdown fins using immuno-histochemistry. In prior studies we found that Acan is initially upregulated in mesenchyme during regeneration (around 3–4 dpa), and later expressed more prominently in the lepidotrichia (around 5 dpa). Upon *hapln1a* knockdown, we observed that Acan protein levels are down regulated in *hapln1a* MO treated fins compared to the control MM treated fins (Fig 5). Next, we determined whether Acan contributes to Hapln1a-Cx43 dependent skeletal phenotypes. We used two independent non-overlapping MOs (one ATG-blocking and one splice-blocking) to knockdown Acan. The effects of *acanb* knockdown were confirmed for both MOs by immuno-staining for Acan. Both MOs caused significant reduction of Acan protein levels (Fig 6A). Together, reduced Acan protein levels following knockdown with *hapln1a*-MO and with two targeting *acanb*-MOs demonstrates that the Acan antibody detects zebrafish Acan protein, although it does not rule out the possibility that the antibody may also detect related zebrafish proteins. We next evaluated the effect of *acanb* knockdown on Hapln1a mediated phenotypes. Knockdown of *acanb* resulted in significant reduction of regenerate length, segment length and cell proliferation (Fig 6B). Similar to *has2* knockdown, the effect of *acanb* knockdown on the level of cell proliferation was not as strong as observed for the *hapln1a* knockdown (see hashed bars in Fig 6B).

Since Acan is important for bone mineralization, we examined bone calcification at 4 dpe using alizarin red, which stains mature bone matrix. The extent of mineralization was determined by calculating the ratio of the total regenerate length to the zone of mineralization (i.e., the length of detectable alizarin red staining). Either the target ATG-blocking MO or the control MM injected side was compared with the respective un-injected side and the percent similarity was determined. In contrast to the control MM treated fins, *acanb* MO treated fins showed a much shorter zone of bone calcification (~20% reduced) (Fig 6C and 6D), suggesting that Acan might play a role in regulating bone matrix maturation and joint formation.

### Hapln1a and Sema3d gene products interact to mediate Cx43 dependent skeletal phenotypes

**Fig 7:**
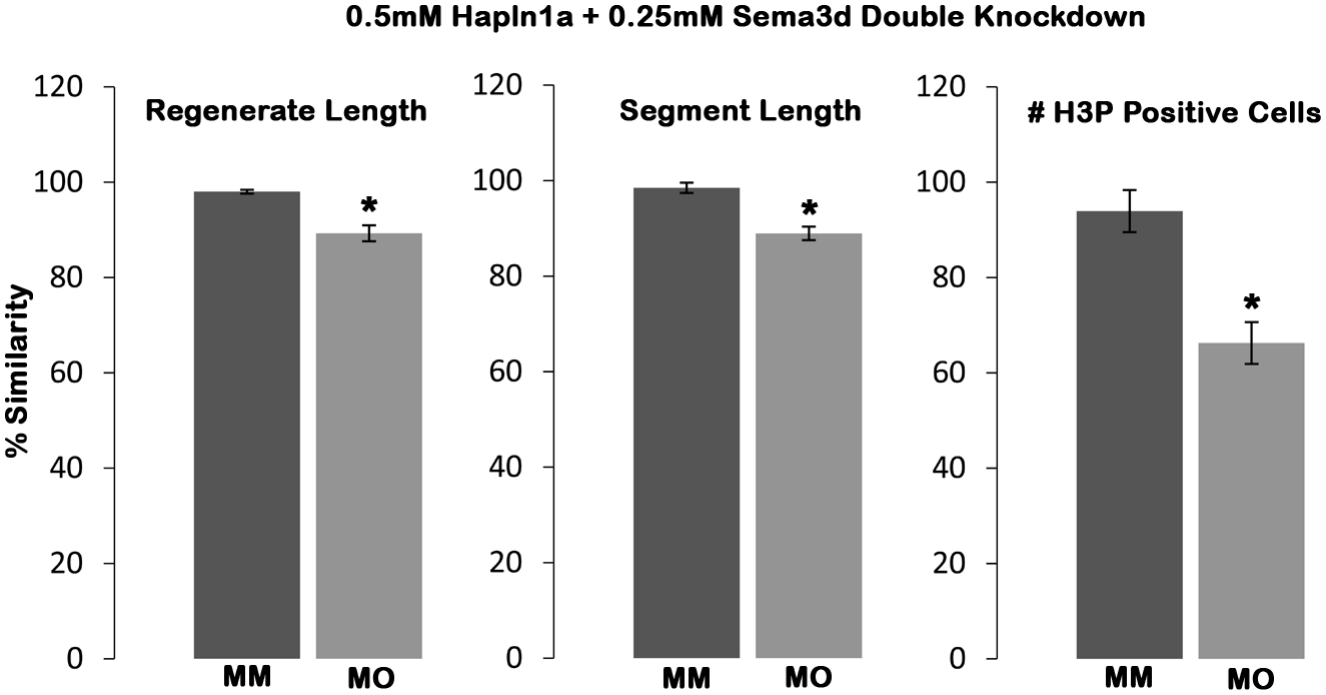
Hapln1a and Sema3d interact genetically to mediate Cx43 phenotypes. Prior to knockdown and electroporation, all fins were amputated at 50% level and allowed to regenerate for 3 days. Fin rays treated with combined targeting MO or combined control MM were measured and compared to their un-injected sides. The ratio of injected (MO or control MO) and un-injected side multiplied by 100 is the percent similarity. Percent similarity greater than 100% reflects the fact that the experimental side can be measurably larger than the control un-injected side. Independent *hapln1a* knockdown at 0.5mM concentration and *sema3d* knockdown at 0.25mM concentration did not produce significant effects on Cx43 dependent phenotypes (not shown). Bar graphs reveal that double knockdown at MO concentrations of 0.5mM Hapln1a and 0.25mM of Sema3d recapitulated the Cx43 knockdown phenotypes (reduced regenerate length, segment length and cell proliferation), suggesting that Hapln1a and Sema3d interact genetically to promote Cx43 function. Students t-test was performed (p<0.05) to determine significance, and the error bars indicate standard error of the mean.

Data from our recent study suggested that *hapln1a* and *sema3d* are transcriptionally independent of each other, meaning that the expression of either does not depend upon the other [16]. However, these findings do not exclude the possibility that the two proteins work together. Several studies demonstrate that GAGs and the ECM function together to regulate signal transduction pathways. For example, GAGs in the ECM play a role during physiological and pathological remodeling processes, such as regeneration, bone development, cancer metastasis and osteoarthritis [44–46]. Moreover, GAGs have been implicated in regulating signaling pathways of molecules such as FGFs, BMPs, Wnts, Hhs and IGFs (reviewed in [47]). Sema3d is a signaling molecule that is known to mediate Cx43 dependent phenotypes [15] and Hapln1a is an ECM protein known to mediate Cx43 dependent phenotypes [16]. Therefore, we wished to test the hypothesis that the protein products function together. To test this hypothesis, we looked for evidence of genetic interaction by performing double knockdowns of *sema3d* and *hapln1a* and testing for synergistic effects. We first identified sub-threshold concentrations for both the *sema3d* and *hapln1a* MOs, where neither single knockdown resulted in a skeletal phenotype. Using MOs validated in our prior studies [16,40], we found that 0.5mM concentration of the *hapln1a* MO and 0.25mM concentration of the *sema3d* MO did not result in skeletal phenotypes (S1 Fig). Next, we performed double knockdowns of both *hapln1a* and *sema3d* using these sub-threshold concentrations (0.5mM Hapln1a MO + 0.25mM Sema3d MO) and compared with the double MM control at the same concentrations. Either the double MO or the double control MM injected side was compared with the respective un-injected side and the percent similarity was determined as described earlier. Interestingly, we observed that the double knockdown resulted in reduced regenerate length (~11% reduced), reduced segment length (~12% reduced) and reduced cell proliferation (~34% reduced) (Fig 7), revealing that *hapln1a* and *sema3d* gene products act synergistically and indeed function in a common molecular pathway.

### Hapln1a-ECM stabilizes Sema3d

**Fig 8:**
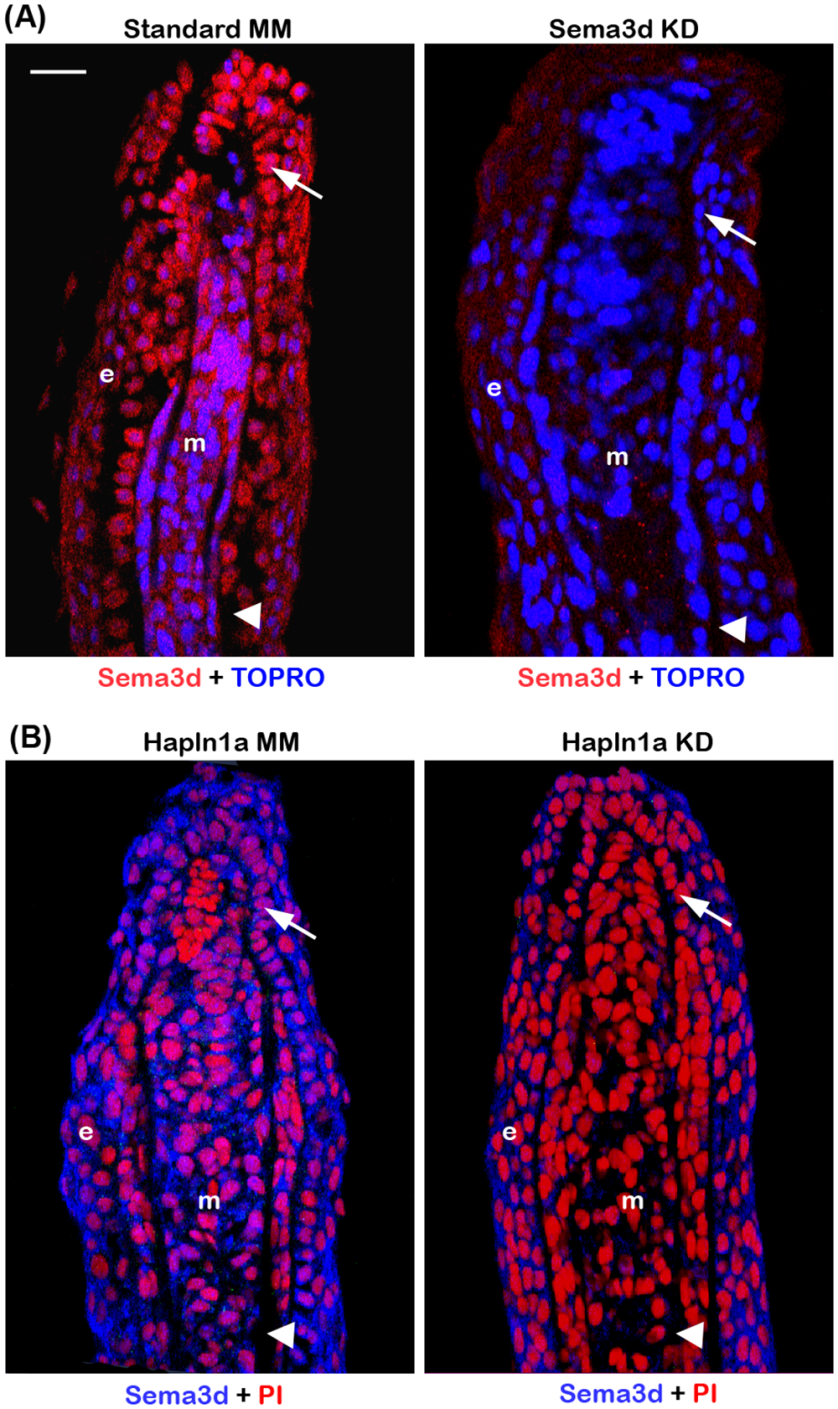
Morpholino mediated knockdown of *hapln1a* results in reduced Sema3d protein levels. (A) Longintudinal section of fin rays treated with control morpholino and *sema3d* targeting morpholino (Sema3d KD). Immuno-staining for Sema3d (red) and counterstained for nuclei with TOPRO (blue). Compared to the control MM treated fins, Sema3d MO treated fins show reduced staining for Sema3d. (B) Longitudinal section of fin rays treated with *hapln1a* control morpholino (Hapln1a MM) and *hapln1a* targeting morpholino (Hapln1a MO). Immuno-staining for Sema3d (blue) and counterstained for nuclei with Propidium Iodide (PI, red). Compared to the control MM treated fins, Hapln1a MO treated fins show reduced staining for Sema3d. Arrow identifies the basal layer of epithelium; arrow head identifies the bone; m, mesenchyme; e, epithelium. Scale bar represents 20 μm.

Based on the genetic interaction data, we hypothesized that Hapln1a-ECM is playing a role in mediating Sema3d dependent signal transduction. It is well known that the ECM can contribute to stabilizing growth factors and thereby influencing growth factor dependent signaling [48]. Hence, it is possible that when Hapln1a protein levels are reduced by *hapln1a* knockdown, the disruption of Hapln1a-ECM affects either the stabilization of Sema3d or affects the presentation of Sema3d to its receptors. Therefore we wished to evaluate Sema3d protein levels. Sema3d protein levels were monitored following Sema3d knockdown to determine if the antibody detected zebrafish Sema3d. Because reduced Sema3d protein levels were observed, this demonstrates that the antibody detects Sema3d (although it does not rule out the possibility that the antibody may also detect related zebrafish proteins) (Fig 8). To determine whether Sema3d stability depends on Hapln1a, we examined the Sema3d protein levels following *hapln1a* knockdown and compared to control MM treated fins by immuno-staining analysis. We observed that Sema3d protein is reduced following *hapln1a* knockdown (Fig 8), suggesting that Hapln1a is required for the stability of Sema3d.

To distinguish the possibility of whether Hapln1a is required for stabilization, or required for both stabilization and presentation of Sema3d to its receptors, we asked if Sema3d overexpression could rescue *hapln1a* knockdown phenotypes. We utilized the transgenic line, *Tg(hsp70*:*sema3d-gfp)* that overexpresses Sema3d-GFP under the control of a heat shock promoter [49]. If Hapln1a is required primarily for the presentation of Sema3d to its receptors, then *hapln1a* knockdown phenotypes will likely not be rescued by overexpression of Sema3d. On the other hand, if Hapln1a dependent ECM is required mainly for Sema3d stabilization, then we expect that *hapln1a* knockdown phenotypes would be rescued by Sema3d overexpression. We first confirmed overexpression of *sema3d-gfp* mRNA and Sema3d-GFP protein in regenerating fins by whole mount ISH for *sema3d* and immuno-staining for Sema3d and GFP on heat shocked versus non-heat shocked *Tg(hsp70*:*sema3d-gfp)* fins. We observed that both *sema3d* mRNA and Seme3d-GFP protein expression was increased in heat shocked fins compared with fins not treated with heat shock (S2 Fig).

**Fig 9:**
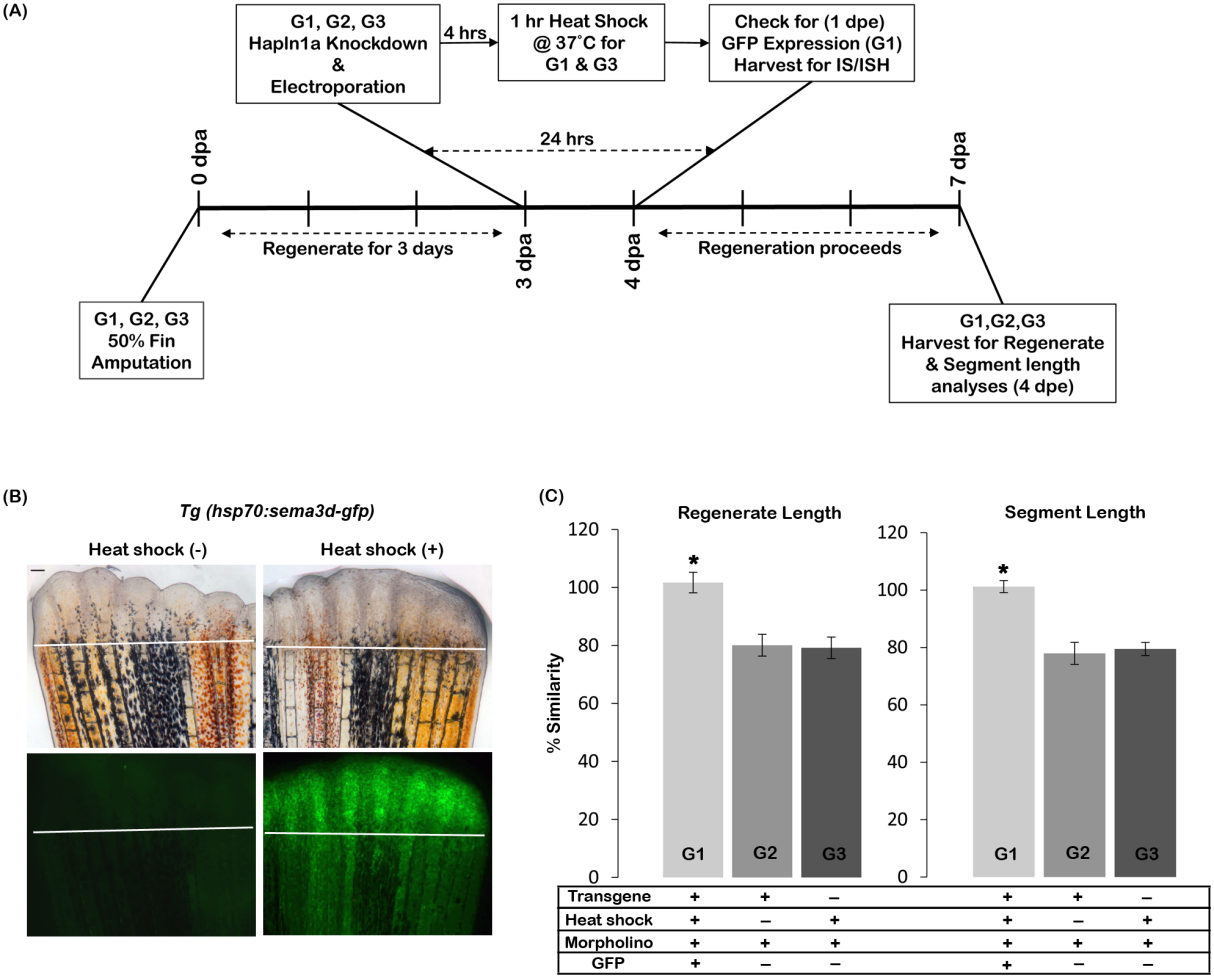
Sema3d overexpression rescues *hapln1a* knockdown phenotypes. (A) Experimental design. Three groups of fish were analysed: G1, G2 and G3. Fins from all three groups were amputated at 50% level. On 3 dpa all animals (G1, G2 and G3) were treated for *hapln1a* knockdown. Four hours post knockdown, G1 and G3 animals were heat shocked for 1hr at 37°C. G2 animals were not treated for heat shock. The following day, 1 dpe, G1 animals were selected for GFP positive fins. For GFP detection, immuno-staining (IS) and *in situ* hybridization experiments (ISH), fins were harvested 1 dpe and for regenerate and segment length analyses fins were harvested at 4 dpe. (B) Heat shock induces Sema3d-GFP (green) expression in *Tg(hsp70*:*sema3d-gfp)*. GFP is not detected in the absence of heat shock. The white line indicates the amputation plane. Scale bar represents 50μm. (C) Following *hapln1a* knockdown the experimental group (G1) that is positive for both transgene and heat shock alone shows rescue for the phenotypes (i.e., shows high percent similarity compared to the un-injected side) whereas the control groups either negative for heat shock (G2) or negative for transgene (G3) fail to show rescue (i.e., shows reduced percent similarity compared to the un-injected side). Students t-test was performed (p<0.05) to determine significance, and the error bars indicate standard error of mean.

The rescue experiment involved 3 different groups of animals (Fig 9A). Group 1 animals (experimental group) were positive for the transgene. This group was treated for *hapln1a* knockdown and Sema3d-GFP was induced 4 hours after knockdown by a 1 hour heat shock at 37°C. Group 2 animals (control 1) were also positive for transgene. This group was also treated for *hapln1a* knockdown, but did not receive the heat shock (and therefore do not overexpress Sema3d). Group 3 animals (control 2) were transgene negative siblings. This group was treated for *hapln1a* knockdown and received 1 hour of heat shock at 37°C. At 24 hours post *hapln1a* knockdown, all animals were screened for GFP expression. Only Group 1 animals showed positive GFP expression in the fin, since they all carried the transgene and were heat shocked for the overexpression of Sema3d (Fig 8B). Only GFP positive fish were selected from Group 1 for further analysis. All three groups were treated for knockdown at 3 dpa. Four hours post knockdown, Groups 1 and 3 were treated for a 1 hour heat shock at 37°C. Fins were harvested from all groups at 4 dpe and regenerate and segment length analyses were completed using the percent similarity method. Therefore, within each group, the injected side was compared with the respective un-injected side and the percent similarity was determined. We found that Group 1 had a high percent similarity (i.e., close to 100%) between the injected vs un-injected side showing that *hapln1a* knockdown phenotypes were fully rescued by overexpression of Sema3d (Fig 9C). We showed that rescue depended on both the heat shock to induce Sema3d overexpression and on the presence of the transgene since neither animals from Group 2 nor animals from Group 3 exhibited rescue of skeletal phenotypes. Thus, for both control groups regenerate length was reduced by ~20% and for segment length, Group 2 showed ~23% reduction and Group 3 showed ~20% reduction (Fig 9C). Therefore, Sema3d overexpression rescues *hapln1a* knockdown phenotypes. These experiments highlight the importance of the Hapln1a-ECM for stabilization of Sema3d, and for Sema3d-based signal transduction.

## Discussion

**Fig 10:**
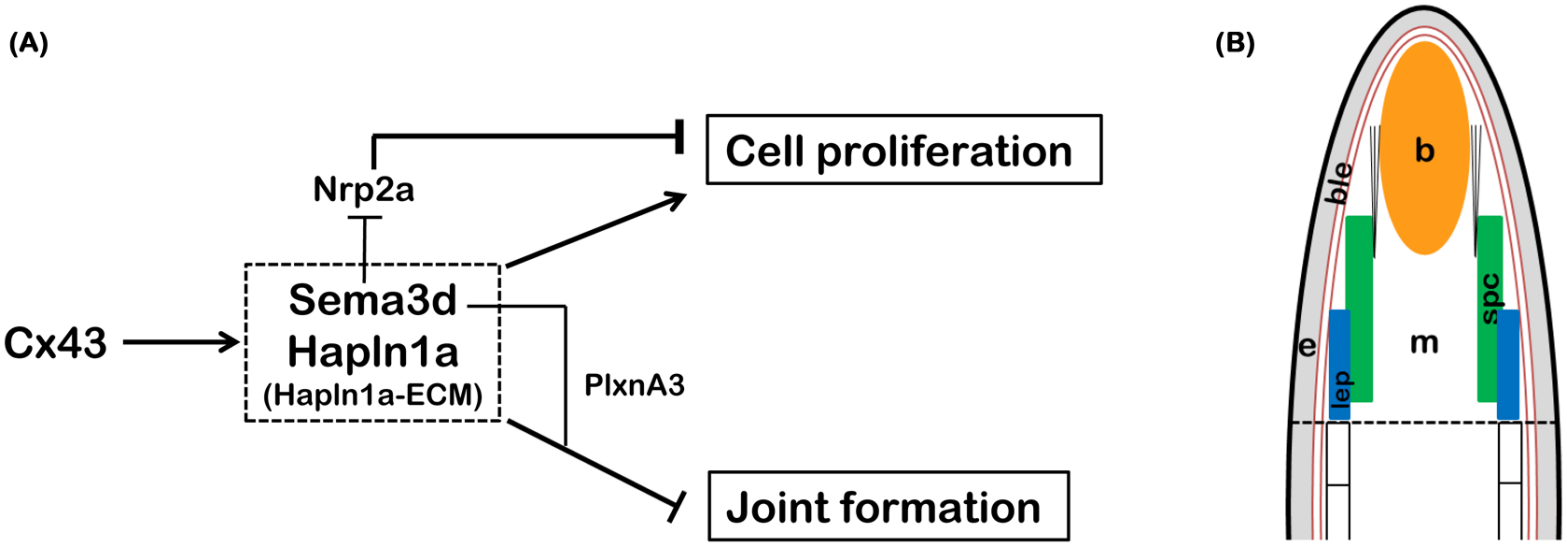
Hapln1a and Sema3d function in a common pathway to mediate Cx43 function during fin regeneration. (A) Proposed pathway showing interactions of Hapln1a-ECM and Sema3d proteins in skeletal growth and patterning. The Hapln1a dependent ECM stabilizes Sema3d protein and permits Sema3d dependent signaling events through its putative receptors, Nrp2a to promote cell proliferation (growth) and PlxnA3 to inhibit joint formation (patterning), to mediate Cx43 functions. (B) Speculative model showing co-operative functioning of Hapln1a-ECM and Sema3d protein. Hapln1a-ECM components HA and Hapln1a are expressed in the blastema (orange), mesenchyme (white), epidermis (grey) and by the skeletal precursor cells (green) in the lateral compartment. Acan is expressed both in the blastema and surrounding the lepidotrichia (blue). The *sema3d* mRNA is produced in the lateral skeletal precursor cells (green) [74], while Sema3d protein is secreted. The Hapln1a-ECM stabilizes the secreted Sema3d protein and may facilitate its proper diffusion to promote signaling events via its receptors (the *nrp2a* mRNA is expressed in the medial mesenchyme while the *plxna3* mRNA is expressed in the lateral skeletal precursor cells, [15]). The black dotted line represents the plane of amputation, b, blastema; ble, basal layer of epithelium; e, epidermis; m, mesenchyme; lep, lepidotrichia; spc, skeletal precursor cells.

Previously we have shown that *hapln1a* knockdown results in significant reduction in HA levels during fin regeneration [16]. Here we tested whether HA is sufficient to cause the observed Hapln1a dependent phenotypes of reduced regenerate length, reduced cell proliferation, and reduced segment length. We found that in addition to HA, Acan also contributes to these phenotypes. Further, we demonstrate the dependence of Sema3d on the presence of the Hapln1a-ECM. We show that Hapln1a and Sema3d genetically interact and we find that *sema3d* overexpression can rescue *hapln1a* knockdown phenotypes. Based on this current study we propose a new model for the Cx43 pathway (Fig 10). Even though transcriptionally *hapln1a* and *sema3d* have been shown to be independent of each other [16], the current study demonstrates that the Hapln1a-ECM plays a role in stabilizing Sema3d protein. Therefore, it is likely that Sema3d signal transduction requires the Hapln1a-ECM. Our findings indicate that the Sema3d and Hapln1a gene products work together to mediate Cx43 phenotypes during growth and patterning in the regenerating fin.

Independent studies in different model systems provide evidence that components of the Hapln1a-ECM (link protein/Hapln, HA, and Acan) are interdependent. HA plays a central role in organizing the ECM where it serves as a central backbone binding to the PGs Acan via the Hapln1 link protein. Moreover, in addition to reduced HA, Has2 deficiency also resulted in reduced Acan content [50]. Conditional knockout of *HAPLN1* (also called *CRTL1*) in the mouse reveals that the function of link protein is indispensable for stabilizing the HA-PG aggregate [24]. Acan deficient mice and chicks are characterized by dwarf limbs, craniofacial abnormalities, and perinatal lethality [51–53]. We have shown that loss of Hapln1a protein is associated with reduced cell proliferation, reduced bony segment length, reduced HA levels [16], and reduced Acan levels (this study). Therefore, the presence of Hapln1a in zebrafish is similarly required for the integrity and composition of the Hapln1a-ECM. Moreover, we demonstrate a requirement for the Hapln1a-ECM during growth and patterning during of the regenerating fin. Future studies will reveal how particular components of the Hapln1a-ECM contribute to specific cellular functions.

HA is ubiquitously present throughout the body and in all the bones and cartilages of the skeleton. HA fulfills various structural and metabolic functions (Reviewed in [54,55]), [50,56]). For example, HA modulates cell adhesion, cell migration, morphogenesis, tumorigenesis, cell survival, apoptosis, and inflammation. However, these biological effects differ depending on HA size. Endogenous high molecular weight HA has been shown to be anti-inflammatory and antiangiogenic [57], while low molecular weight HA can induce angiogenesis and tumor progression [58]. The three HA synthases Has1, Has2 and Has3 reside on the plasma membrane and extrude HA directly into the extracellular space [59–63]. All three are integral membrane proteins with six putative transmembrane domains [63], and all contain conserved amino acid residues that have both GlcNAc and GlcA transferase activity [64]. The Has enzymes each synthesize HA of similar composition but may differ in the rate of synthesis, chain length and the ease with which HA is released from the cell surface [41], although it is not clear how differences in these activities influence the function of HA. However, of the three enzymes, *HAS2* is uniquely essential for life and neither Has1 nor Has3 compensate for the loss of *HAS2* [42]. Conditional *HAS2* gene knockout in mice causes shortened axial and appendicular skeleton and defects in patterning of digits [50,65], and strikingly, overexpression of Has2 in the developing chick limb bud mesoderm also leads to shortened bones with abnormal morphology and positioning [56]. Together, these findings suggest a critical need for regulating HA metabolism and highlight the significance of HA produced by Has2. Our findings that *has2* knockdown, but not *has1* knockdown, influences regenerate length and segment length, also suggests that HA synthesized by Has2 could be functionally distinct, consistent with these prior studies.

During fin regeneration, reduced HA exhibited a stronger effect on cell proliferation than on patterning. Previously, we found that HA is expressed in a conserved pattern during fin regeneration [37]. From 3–5 dpa, HA is strongly upregulated in the distal stump and proximal blastema (i.e., just above and below the amputation plane) compared to the distal end of the regenerate, where it may maintain the proliferative state of the mesenchymal cells by limiting contact inhibition between cells that leads to tissue differentiation [66–70]. Indeed, lower levels of HA resulting from reduced Hapln1a [16], reduced *has1*, or reduced *has2*, all reduce the level of cell proliferation.

The ECM is no longer considered to be an inert substance surrounding cells, but rather, can contribute to the regulation of multiple signaling pathways [71]. For example, the ECM can serve as a reservoir for secreted growth factors. Local release of ECM-bound growth factors could influence temporal regulation of signal transduction pathways, and/or the half-life of secreted growth factors. Moreover, the concentration of signaling molecules at a particular location may contribute to the establishment of morphogen gradients that play critical roles during patterning and developmental processes [72,73]. Our findings reveal that the Hapln1a-ECM is crucial for the stability of the secreted growth factor, Sema3d. Further work is required to understand how Sema3d interacts with components of the Hapln1a-ECM, and how these interactions may regulate Sema3d-receptor based signal transduction.

## Supporting Information

S1 Fig Knockdown of *hapln1a* at 0.5mM concentration and *sema3d* at 0.25mM concentration did not produce significant effects on Cx43 dependent phenotypes. Bar graphs show the effect of *hapln1a* MO at 1mM, 0.75mM and 0.5mM concentrations and *sema3d* MO at 1mM, 0.75mM, 0.5mM and 0.25mM concentrations. Compared to 1mM MM control treated fins, *hapln1a* knockdown at 0.5mM and *sema3d* knockdown at 0.25mM concentration did not have a significant effect on regenerate and segment length.
(TIF)

S2 Fig Heat shock induces upregulation of *sema3d* mRNA and Sema3d protein in *Tg(hsp70*:*sema3d-gfp)*. (A) Whole mount in situ hybridization shows increased expression of *sema3d* mRNA in heat shock treated fins compared to the untreated fins. Black line indicates amputation plane. (B) Immuno-staining analysis of longitudinal fin sections reveal increased expression of GFP (green) and Sema3d (Red) in heat shock treated fins compared to the untreated fins. DAPI (blue) is used as the counter stain and stains the nuclei. Arrows indicate basal layer of epithelium and arrow head marks the bone. e, epidermis; m, mesenchyme. Scale bar represents 100μm in both panels.
(TIF)
