# Supplementary figures and images for: Cx43-Dependent Skeletal Phenotypes Are Mediated by Interactions between the Hapln1a-ECM and Sema3d during Fin Regeneration
Source: PLoS One. 2016 Feb 1;11(2):e0148202. doi: 10.1371/journal.pone.0148202 (PMC4734779; doi:10.1371/journal.pone.0148202)

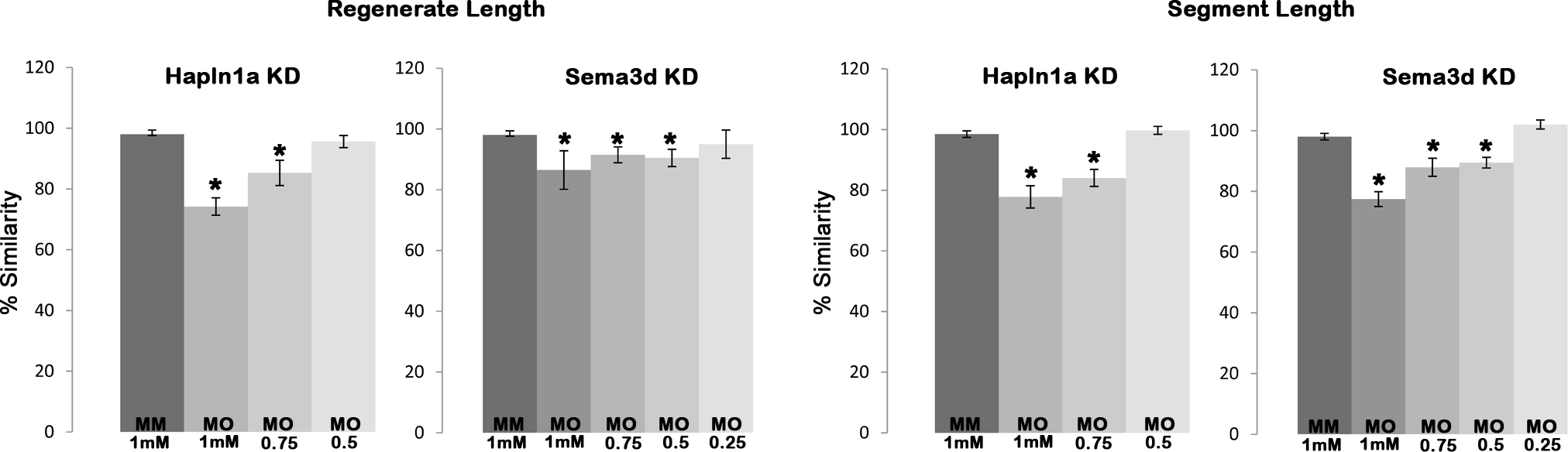

Supplement: S1 Fig — Bar graphs show the effect of hapln1a MO at 1mM, 0.75mM and 0.5mM concentrations and sema3d MO at 1mM, 0.75mM, 0.5mM and 0.25mM concentrations. Compared to 1mM MM control treated fins, hapln1a knockdown at 0.5mM and sema3d knockdown at 0.25mM concentration did not have a significant effect on regenerate and segment length. (TIF) [file pone.0148202.s001.tif]

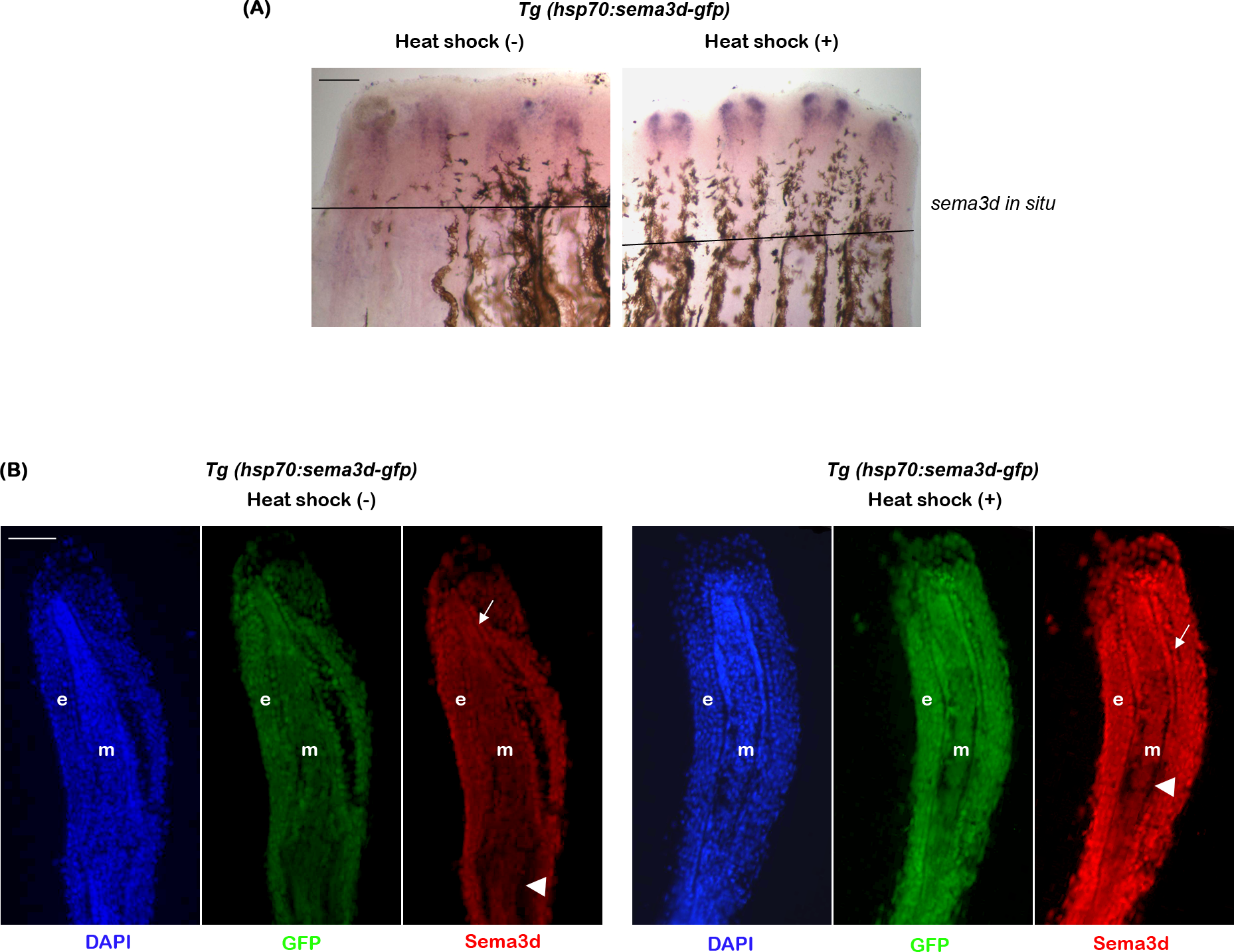

Supplement: S2 Fig — (A) Whole mount in situ hybridization shows increased expression of sema3d mRNA in heat shock treated fins compared to the untreated fins. Black line indicates amputation plane. (B) Immuno-staining analysis of longitudinal fin sections reveal increased expression of GFP (green) and Sema3d (Red) in heat shock treated fins compared to the untreated fins. DAPI (blue) is used as the counter stain and stains the nuclei. Arrows indicate basal layer of epithelium and arrow head marks the bone. e, epidermis; m, mesenchyme. Scale bar represents 100μm in both panels. (TIF) [file pone.0148202.s002.tif]
